# Dual RNA Sequencing of *Vitis vinifera* during *Lasiodiplodia theobromae* Infection Unveils Host–Pathogen Interactions

**DOI:** 10.3390/ijms20236083

**Published:** 2019-12-03

**Authors:** Micael F. M. Gonçalves, Rui B. Nunes, Laurentijn Tilleman, Yves Van de Peer, Dieter Deforce, Filip Van Nieuwerburgh, Ana C. Esteves, Artur Alves

**Affiliations:** 1 Department of Biology, CESAM, University of Aveiro, 3810-193 Aveiro, Portugal; mfmg@ua.pt (M.F.M.G.); r.nunesbarros@ua.pt (R.B.N.); 2 Laboratory of Pharmaceutical Biotechnology, Campus Heymans, Ottergemsesteenweg 460, B-9000 Ghent, Belgium; Laurentijn.Tilleman@ugent.be (L.T.); dieter.deforce@ugent.be (D.D.); Filip.VanNieuwerburgh@ugent.be (F.V.N.); 3 Department of Plant Biotechnology and Bioinformatics, Ghent University, 9052 Ghent, Belgium; yves.vandepeer@psb.vib-ugent.be; 4 Center for Plant Systems Biology, VIB, 9052 Ghent, Belgium; 5 Department of Biochemistry, Genetics and Microbiology, University of Pretoria, Pretoria 0028, South Africa; 6 Faculty of Dental Medicine, Center for Interdisciplinary Research in Health (CIIS), Universidade Católica Portuguesa, Estrada da Circunvalação, 3504-505 Viseu, Portugal; acesteves@viseu.ucp.pt

**Keywords:** dual RNA-Seq, grapevine, botryosphaeria dieback, plant defense, pathogenesis

## Abstract

*Lasiodiplodia theobromae* is one of the most aggressive agents of the grapevine trunk disease Botryosphaeria dieback. Through a dual RNA-sequencing approach, this study aimed to give a broader perspective on the infection strategy deployed by *L. theobromae*, while understanding grapevine response. Approximately 0.05% and 90% of the reads were mapped to the genomes of *L. theobromae* and *Vitis vinifera*, respectively. Over 2500 genes were significantly differentially expressed in infected plants after 10 dpi, many of which are involved in the inducible defense mechanisms of grapevines. Gene expression analysis showed changes in the fungal metabolism of phenolic compounds, carbohydrate metabolism, transmembrane transport, and toxin synthesis. These functions are related to the pathogenicity mechanisms involved in plant cell wall degradation and fungal defense against antimicrobial substances produced by the host. Genes encoding for the degradation of plant phenylpropanoid precursors were up-regulated, suggesting that the fungus could evade the host defense response using the phenylpropanoid pathway. The up-regulation of many distinct components of the phenylpropanoid pathway in plants supports this hypothesis. Moreover, genes related to phytoalexin biosynthesis, hormone metabolism, cell wall modification enzymes, and pathogenesis-related proteins seem to be involved in the host responses observed. This study provides additional insights into the molecular mechanisms of *L. theobromae* and *V. vinifera* interactions.

## 1. Introduction

Grapevine (*Vitis vinifera*) is widely cultivated and an economically important fruit crop worldwide [1]. Diseases of fungal origin such as grapevine trunk diseases (GTDs) are a significant factor limiting grapevine productivity and longevity [2,3]. Botryosphaeria dieback caused by the fungi of the family *Botryosphaeriaceae* that grow primarily in mature wood causes dieback as a consequence of the development of a necrotic wood canker/lesion [3]. Botryosphaeria dieback leads to a loss of productivity, reducing profit and longevity [4]. Some authors suggest that environmental changes, such as drought and increase of temperature, may induce some fungi to become aggressive pathogens, killing their hosts through the release of cell wall degrading enzymes, inhibitory proteins, and toxins [5,6,7,8].

*Lasiodiplodia theobromae* is a common phytopathogenic fungus and one of the most aggressive species found in grapevines [1,9,10,11]. It is mostly found in tropical and subtropical regions [12,13] and has an optimal temperature range of 27 °C–33 °C [14]. This pathogen has a great adaptation capacity and has been associated with numerous hosts and diseases [13]. Although it is more frequent in grape-growing regions with high temperatures and low precipitation [9,10,11], it has also been reported in temperate climates [15,16,17,18].

The interaction between plants and their pathogens is a dynamic and complex process. These interactions should be analyzed as a duel process, and the plant’s reactions should not be separated from the ones of the pathogen [19]. When a pathogen interacts with its host, it will trigger a complex host defense response activating various processes, such as penetration resistance, recognition by pattern recognition receptors (PRRs), phytohormone signaling pathways, secretory pathways, and secondary metabolite production [20]. RNA sequencing (RNA-Seq) is a powerful technology that has been widely implemented to investigate host defense mechanisms during infection. So far, this approach has been applied mainly to the host or to the pathogen separately [19]. Dual RNA sequencing allows the study of both host and pathogen transcriptomes simultaneously, detecting pathogen-specific transcripts in the same sample, providing a more complete insight into the pathogen infection biology and host defense mechanisms [19,21,22]. This approach has already been applied in a few studies of plant–pathogen interactions in crops [23,24,25,26] and trees [20,27,28].

Using a dual RNA-Seq approach, we aimed to identify the pathogenicity factors produced by *L. theobromae* and determine the *V. vinifera* defense responses to the pathogen. Overall, we intended to contribute to unraveling host–pathogen interactions and provide helpful information for the future development of strategies of disease control and management.

## 2. Results

### 2.1. Macroscopic Analysis

**Figure 1:**
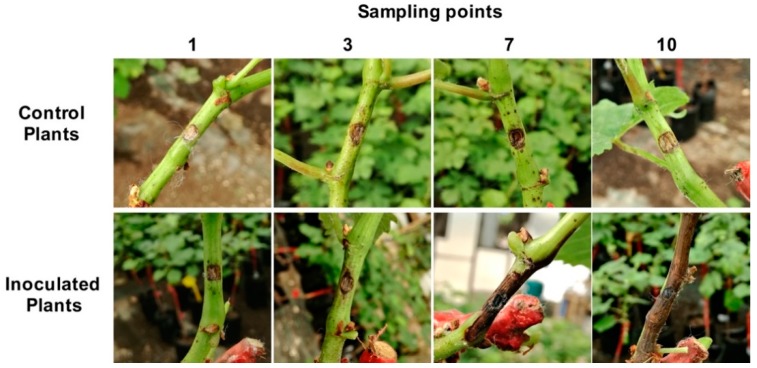
Symptom development in *V. vinifera* following *L. theobromae* inoculation for 1, 3, 7, and 10 days post-inoculation.

Progression of necrosis was observed throughout the duration of the experiment (Figure 1). No lesions beyond the wound site were observed in “mock” inoculated plants in all sampling points. At 1 day post-inoculation (dpi), no lesions related to fungal infection were observed. After 3 dpi, a slight browning around the inoculation sites in inoculated plants was observed (0.5 ± 0.1 cm). At 7 dpi, the lesion in infected plants progressed (5 ± 0.2 cm), and at 10 dpi, the lesion length was intensified, covering 9 ± 0.2 cm in length.

### 2.2. Dual RNA-Seq Analysis

To characterize the responses of *V. vinifera* to *L. theobromae* infection in the green shoots, and simultaneously to characterize gene expression of *L. theobromae* during the process, we profiled both transcriptomes using a dual RNA-Seq approach at the four time points described above. Reads were mapped to the combined reference genomes of grapevine (*V. vinifera* var. “PN40024”) and of *L. theobromae* (NCBI SAMN08892999). Considering all samples, 15 ± 4 million reads were mapped to the *V. vinifera* genome. Considering the infected samples, 11 ± 7 thousand reads were mapped to the *L. theobromae* genome. Saturation curves (Figure S1) show that most genes of the *V. vinifera* genome were sequenced in all the samples, representing an adequate library complexity and sequencing depth. Saturation was not achieved for *L. theobromae* genes in the infected samples (Figure S2), i.e., we have not sequenced enough to discover all the *L. theobromae* genes. However, this does not mean that no differential expression gene analysis can be performed. A total of 23,417 and 7115 genes were identified for *V. vinifera* and *L. theobromae*, respectively. Only genes present in at least three replicates with a minimum of one count per million (cpm) were used for differential expression analysis. In this way, 18,062 and 4095 genes of *V. vinifera* and *L. theobromae*, respectively, were considered as quantifiable genes.

### 2.3. Differentially Expressed Genes (DEGs) in V. vinifera

**Figure 2:**
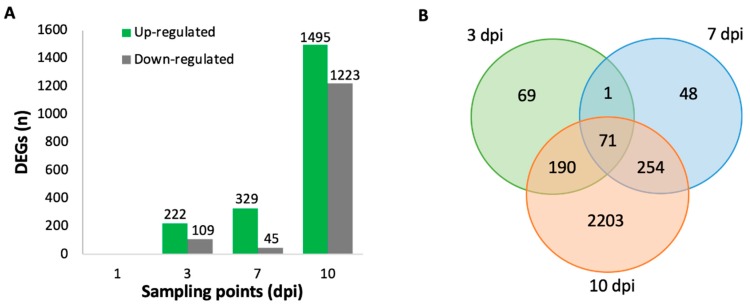
(**A**) Number of up-regulated and down-regulated significantly differentially expressed genes (false discovery rate, FDR <0.01 and a log_2_ fold change (FC) >1 or < −1) in *V. vinifera* after inoculation with *L. theobromae* compared to control for 1, 3, 7, and 10 days post-inoculation. Only genes present in all replicates were taken into account. (**B**) Venn diagram of differentially expressed genes among the three time points (3 vs. 7 vs. 10 dpi).

Differential gene expression analysis of *V. vinifera* inoculated with *L. theobromae* compared to control resulted in a total of 0, 331, 374, and 2718 DEG at 1, 3, 7, and 10 dpi, respectively (Figure 2A). For each time point, there was a higher number of up-regulated genes than down-regulated genes. The most extensive transcriptional reprogramming caused by infection was observed in stems at 10 dpi. A total of 69, 48, and 2203 DEGs were specific for 3, 7, and 10 dpi, respectively, while 71 DEG were common to all sampling points (Figure 2B). To associate the DEGs to a function, the sequence of the DEGs were BLASTed against the UniProtKB/Swiss-Prot database with an e-value of 10^E−10^ (Tables S1–S3) [29,30].

### 2.4. Predominant Functions (GO) of V. vinifera DEGs during L. theobromae Infection

**Figure 3:**
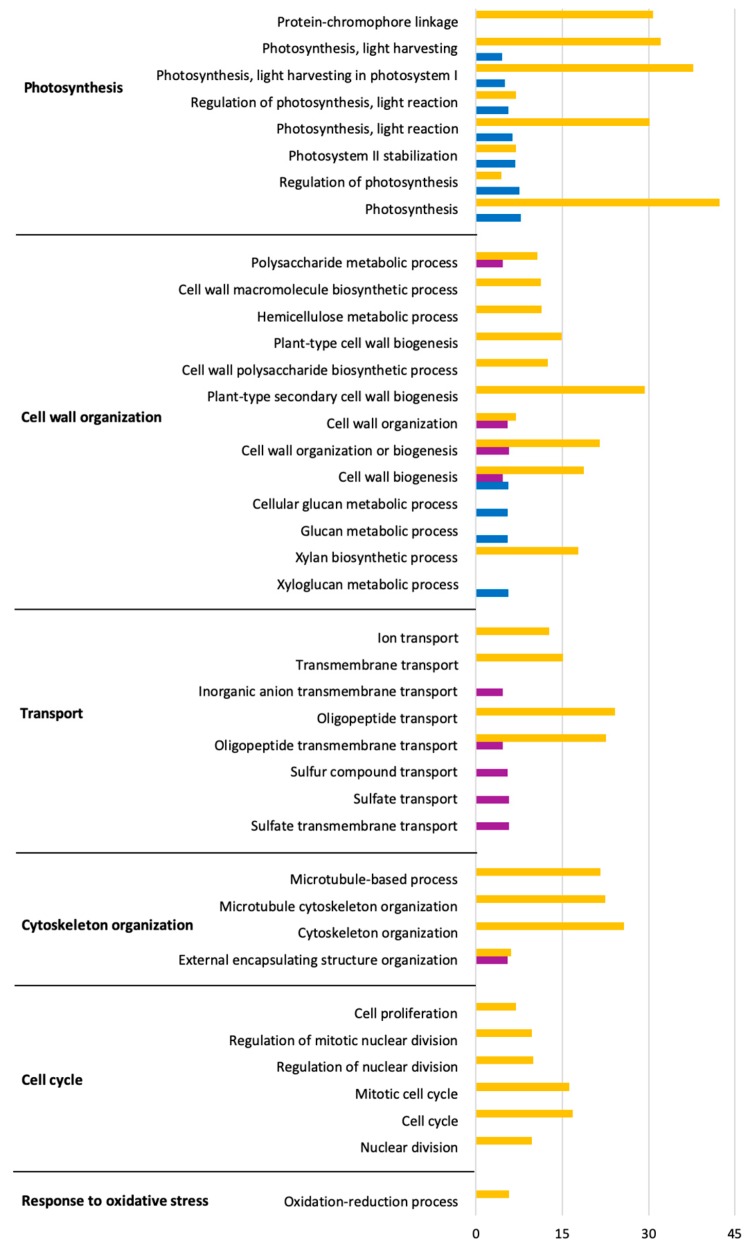
Gene Ontology (Biological process) enrichment analysis of down-regulated genes in response to *L. theobromae* inoculation in *V. vinifera* at 3 (blue), 7 (purple), and 10 (orange) dpi. The *x*-axes represent the absolute log_2_(q-value) based on hypergeometric distribution followed by false discovery rate correction (FDR < 0.05).

**Figure 4:**
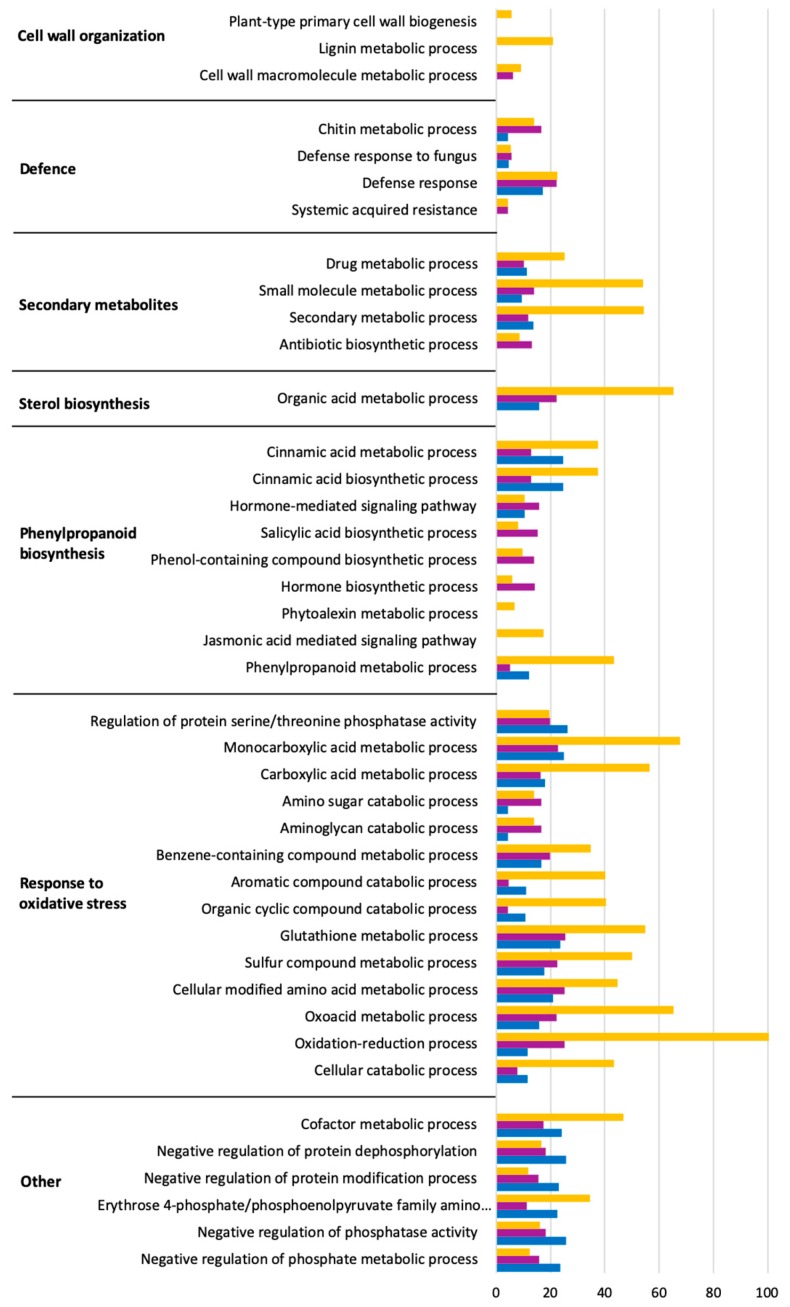
Gene Ontology (Biological process) enrichment analysis of up-regulated genes in response to *L. theobromae* inoculation in *V. vinifera* at 3 (blue), 7 (purple), and 10 (orange) dpi. The *x*-axes represent the absolute log_2_(q-value) based on hypergeometric distribution followed by false discovery rate correction (FDR < 0.05).

The functions of the DEGs based on Gene Ontology (GO) analysis are listed in Tables S1–S3. Significant biological processes were obtained via GO terms enrichment analysis. Up- and down-regulated genes enriched in each GO term are shown in Figure 3 and Figure 4 within the significantly affected (false discovery rate, FDR) < 0.05). The complete list can be found in Table S4. The results showed that *L. theobromae* affected several genes down-regulated in *V. vinifera* involved in photosynthesis, cell wall and cytoskeleton organization, transport, and cell cycle. Most of the enriched terms of up-regulated genes are involved in defense response, secondary metabolites, sterol and phenylpropanoid biosynthesis, and response to oxidative stress.

### 2.5. Time Series Analysis of V. vinifera Genes

**Figure 5:**
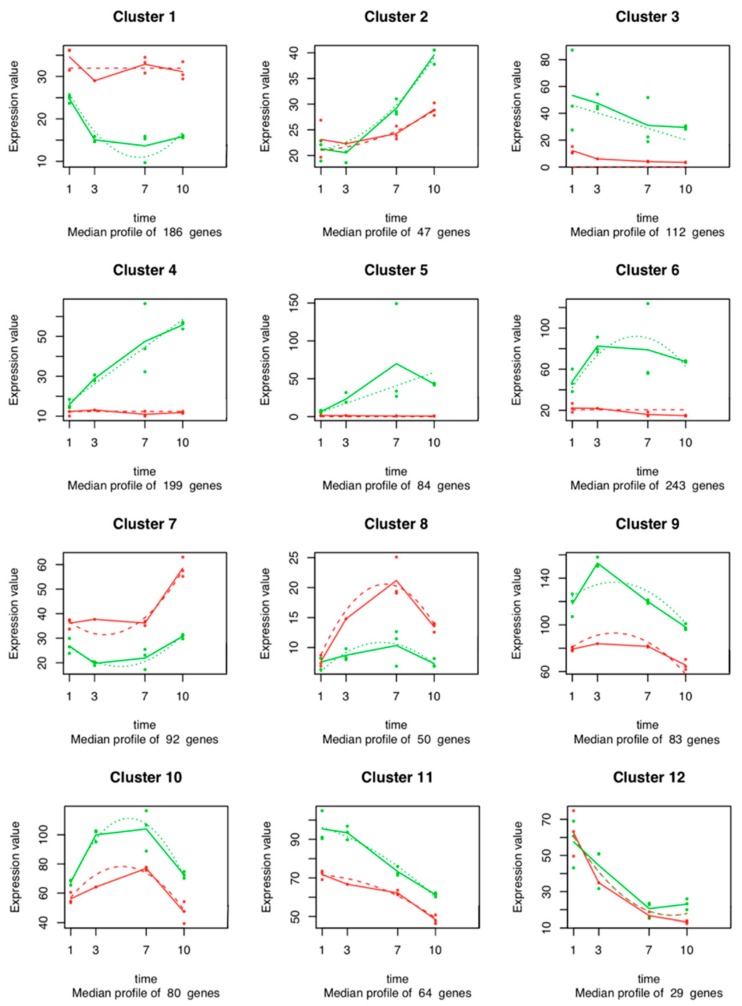
Expression profiles of the *V. vinifera* gene clusters sharing a similar expression pattern during the infection of grapevine stems. In each graph, the dots show the median expression value (i.e., normalized count) of the genes in the cluster for each biological replicate at each time point. The solid red (control group) and green (inoculated plants) lines connect the median of these median expression values at each time point. Dashed lines represent the fitted regression model that was performed on each cluster, modeling the relationship between median expression values of the cluster and the time points.

A total of 1269 out of the 12,841 temporal DEGs in the time series analysis of the *V. vinifera* genes was fitted with a regression model with an R^2^ > 0.8 and were used for cluster analysis. Twelve clusters were defined (Figure 5, Table S5). Each cluster contains genes that have similar expression profiles following the same trend along time. Clusters 3, 4, 5, 6, 9, 10, and 11 (with 112, 199, 84, 243, 83, 80, and 64 genes, respectively) contain transcripts up-regulated in the inoculated plants at all time points. These transcripts encode glutathione transferases (GSTs), cytochrome P450 oxygenases, pleiotropic drug resistance (PDR) transporters, pathogenesis-related (PR) proteins, thaumatin-like proteins (TLPs), stilbene synthase (STS), chalcone synthase (CHS), phenylalanine ammonia lyase (PAL), flavonoid biosynthesis, calmodulin-like proteins, endochitinases, and pectinesterases. On the other hand, Clusters 1, 7, and 8 (with 186, 92, and 50 genes) contain fewer abundant genes in the inoculated plants at all time points. The expression of genes in cluster 1 decreases from Days 1 to 7 in the inoculated plants, and it increases between 7 and 10 dpi. Cluster 1 genes exhibit the same expression level in the control conditions. Cluster 2 shows genes whose expression increases over time, both in the inoculated plants and in the control plants. However, the median expression of these genes in the inoculated plants is clearly overtaking the expression of these genes in the control plants after 3 dpi. Clusters 3, 11, and 12 include genes whose expression decreases over time, both in inoculated and in control plants. The expression of genes in Cluster 4 increases through time in the inoculated plants but remains stable in control conditions. Cluster 5 shows genes whose expression increases along time in inoculated plants, while there was no expression in control plants. The expression of genes in Cluster 6 increases during time but decreases from Day 7 until the end of the experiment in inoculated plants. In control plants, the genes remained at the same expression level. Cluster 7 includes genes whose expression increases only after 7 dpi both in inoculated and control plants, while the expression of genes in Cluster 8 increases over time and decreases from Day 7. The expression of genes in Clusters 9 and 10 increases until Day 3 but show a decrease between 3 and 7 dpi in both conditions.

### 2.6. Inducible Defence Mechanisms during L. theobromae Infection

One of the noteworthy aspects of the RNA-Seq data obtained was that several PR genes were particularly differentially expressed—mostly up-regulated—with high fold-change values (Tables S1–S3 and S5). The most prominent PR genes were PR-3 (chitinase I and IV), PR-4 (chitin-binding) (e.g., *Chi4C*, *VIT_15s0046g01590*, *VIT_15s0046g01600*, *VIT_05s0094g00330*, *VIT_11s0149g00280*, *VIT_05s0094g00260*, and *VIT_14s0081g00050*), PR-5 (thaumatin-like) (e.g., *VIT_02s0025g04300*, *VIT_13s0019g02020*, *VIT_14s0066g02130*, and *VIT_17s0000g02470*), and PR-10 (ribonuclease-like) (e.g., *PR10.2*, *PR10.3*, *VIT_05s0077g01530*, *VIT_05s0077g01540*, *VIT_05s0077g01550*, *VIT_05s0077g01560*, *VIT_05s0077g01600*, *VIT_05s0077g01690*, and *VIT_01s0011g05150*). PR-12 (defensin-like) were also observed (*VIT_19s0085g00610*). We also observed the up-regulation of a hypothetical endo-1,3(4)-β-glucanase 2 gene (*VIT_14s0083g00340*) (Cluster 6, Figure 5).

A set of PDR transporters genes (e.g., *VIT_09s0002g05530*, *VIT_09s0002g05560*, *VIT_09s0002g05570*, and *VIT_09s0002g05590*) that are responsible for the transportation of molecules through the membrane in response to stress were also induced.

Among the activation of defense mechanisms induced by infection, genes encoding GTS (e.g., *VIT_08s0040g00920*, *VIT_05s0049g01090*, and *VIT_05s0049g01100*, which are involved in multiple disease resistance) and cytochrome P450 (e.g., *VITISV_011074*, involved in oxidation of the plant hormone jasmonoyl-L-isoleucine; *VITISV_033534*, involved in ginsenoside biosynthesis; *VIT_03s0063g01480*, involved in methoxsalen biosynthesis; *VIT_07s0141g00890* involved in catalysis of various fatty acids) were also observed to be up-regulated.

### 2.7. DEGs Involved in ET and JA Biosynthesis and Ca^2+^ Signaling in the Inoculated Green Shoots

Several DEGs encoding Ethylene/Jasmonic acid (ET/JA) signaling pathways such as genes related to phytoalexin biosynthesis, such as PAL (e.g., VIT_16s0039g01100, VIT_16s0039g01110, VIT_16s0039g01130, VIT_16s0039g01170, VIT_16s0039g01240, VIT_00s2849g00010, VIT_13s0019g04460, and VIT_06s0004g02620), STS (e.g., VIT_16s0100g00750, GSVIVT00007357001, VIT_16s0100g00920, VIT_16s0100g00830, VIT_16s0100g00850, VITISV_020801, VIT_16s0100g00880, VIT_16s0100g00900, GSVIVT00004047001, VIT_16s0100g01000, VIT_16s0100g01020, VIT_16s0100g01040, VITISV_026018, VIT_16s0100g01120, RS2, VIT_16s0100g01140, VIT_16s0100g01150, and VIT_16s0100g01170) and CHS (e.g., CHI1, CHS, CHS3, and VIT_14s0068g00920) were identified. The expression data showed that the genes involved in ET/JA biosynthesis were rapidly induced upon infection (e.g., Clusters 3 and 6, Figure 5).

Genes related to calcium (Ca^2+^) signaling, such as genes related to Ca^2+^-dependent protein kinases (CDPKs), were identified (*VIT_08s0032g01220*). In addition, we identified genes encoding leucine-rich repeat (LRR) receptor-like kinases (RLKs) up- and down-regulated. These receptors with receptor-like serine/threonine-protein kinases (STKs) belong to the pattern recognition receptors (PRRs) family (e.g., *VIT_19s0014g04130*, *VIT_17s0000g06710*, *VIT_07s0104g00850*, *VIT_00s0220g00120*, *VIT_07s0129g00960*, *VIT_08s0058g00540*, *VIT_08s0056g00150*, *VIT_08s0058g01340*, *VIT_08s0040g00060*, *VIT_10s0003g00330*, *VIT_10s0003g05160*, *VIT_11s0016g05720*, *VIT_12s0035g02090*, and *VIT_14s0128g00160*). The activation of PRRs is the first step to initiate the innate immune mitogen-activated protein (MAP) kinase signaling cascade, resulting in enhanced resistance against pathogens [31]. A decrease in the expression through time of the *VIT_17s0000g01970* gene (uncharacterized protein with MAP kinase activity) was observed (Cluster 3, Figure 5). Genes encoding NADPH oxidases respiratory burst oxidase homologs (Rboh) were also activated (*VIT_11s0016g00540*, *VIT_02s0025g00510*, and *VIT_14s0060g02320*).

Calmodulin/Calmodulin-like (CaM/CML) transcripts were also identified (e.g., VIT_08s0056g00290, VIT_01s0010g02930, VIT_01s0010g02940, VIT_01s0010g02950, VIT_01s0010g02970, VIT_01s0010g03040, VIT_01s0010g03000, VITISV_011291, VITISV_011291, VIT_14s0171g00150, VIT_18s0122g00180, VIT_18s0001g01630, VITISV_006244, VITISV_041123, VIT_05s0102g00450, and VIT_06s0080g00450). Genes of the WRKY family transcription factor, which play a key role in the regulation of disease resistance, were also represented in this set of signaling genes (VIT_08s0058g00690 and VIT_06s0004g07500).

### 2.8. Predominant GO Functions of L. theobromae DEGs in the Time Series Analysis

**Figure 6:**
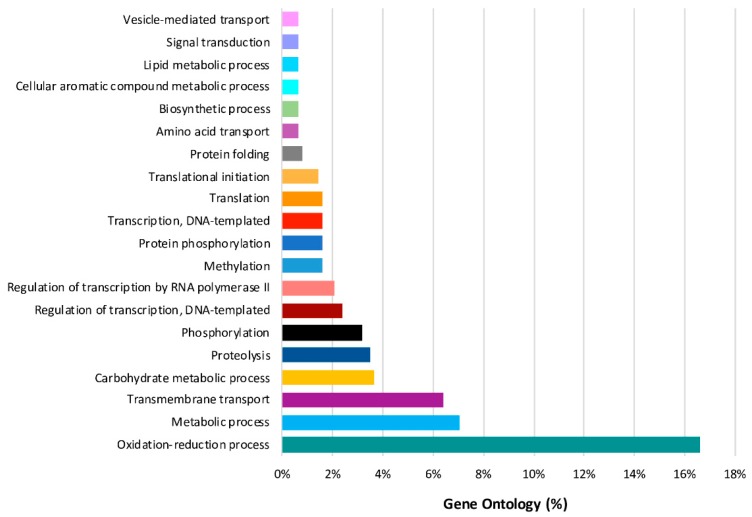
Gene Ontology terms (biological process) of the differentially expressed genes of *L. theobromae* over time.

**Figure 7:**
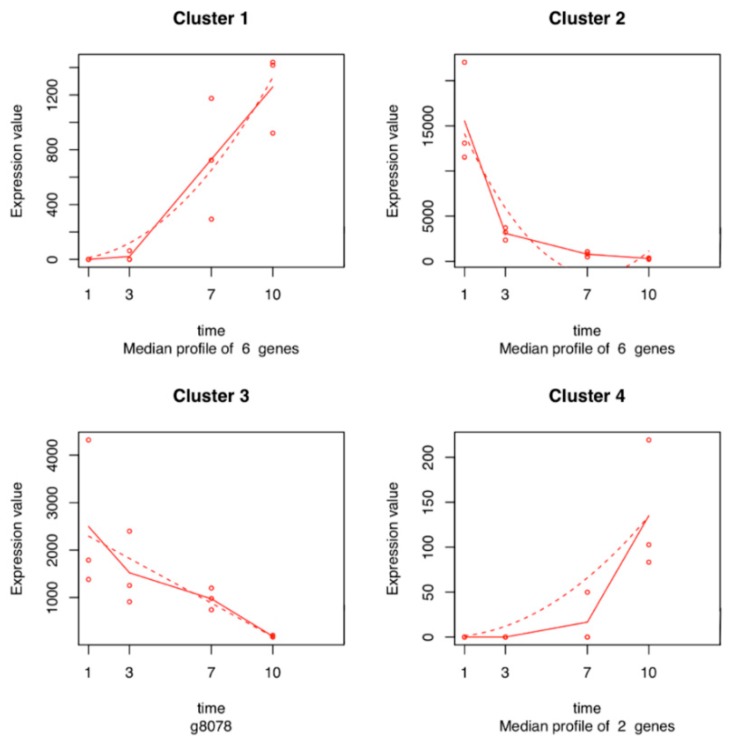
Expression profiles of *L. theobromae* gene clusters sharing a similar expression pattern during the colonization of grapevine stems. In each graph, the dots show the median expression value (i.e., normalized count) of all the genes in the cluster for each biological replicate at each time point. The solid lines represent the median of these median expression values at each time point. Dashed lines represent the fitted regression model that was performed on each cluster, modeling the relationship between median expression values of the cluster and the time points.

Seven hundred and six genes were differentially expressed in the time series analysis of *L. theobromae*. The functions of these temporal DEGs over time, based on GO analysis, are listed in Table S5. The best represented functions are related to processes involved in oxidation reduction (17%), metabolism (7%), transmembrane transport (6%), carbohydrate (4%), and proteolysis (4%) (Figure 6). Fifteen of the 706 temporal DEGs in the time series analysis of the *L. theobromae* genes were fitted with a regression model with an R^2^ >0.8 and were used for cluster analysis. Four clusters were defined (Figure 7 and Table S6). Each cluster contains genes that have similar expression profiles following the same trend over time. Clusters 1 and 4 (with six and two genes, respectively) show an increase in gene expression over time. These genes are mainly related to fungal development. Clusters 2 and 3 include six and one genes, respectively, and their expression decreases throughout time.

Twenty genes encoding cell wall-degrading enzymes (CWDEs) were identified differentially expressed in the transcriptome of *L. theobromae*: pectinases such as pectate lyases (*BK809_0005263*, *BK809_0000650*, *BKCO1_1000601*, and *UCDDS831_g08741*) and glycoside hydrolases (GHs) families 1, 2, 5, 10, 28, 29, 42, 43, 61, and 79 (e.g., *BKCO1_6100046*, *BKCO1_940007*, *BKCO1_1000349*, *BKCO1_1000574*, *BKCO1_1800061*, *BKCO1_2800065*, *UCDDS831_g08450*, *MPH_05314*, *MPH_03522*, *MPH_08407*, and *BKCO1_2500059*). Genes encoding a polygalacturonase (*BK809_0000362*), xylanases (*UCRNP2_6170*, *UCRNP2_4063*), and a pectinesterase (*UCRNP2_4383*) were also identified in the *L. theobromae* dataset and have been associated with pectin degradation. Several genes involved in the secondary metabolism such as cytochrome P450 genes were well represented in the transcriptome of *L. theobromae* (e.g., *UCRNP2_7215*, *BKCO1_16000171*, *BKCO1_9000170*, *UCRNP2_1268*, *BKCO1_8400022*, *UCDDS831_g08095*, *BKCO1_1000650*, *BKCO1_27000127*, *BKCO1_4700086*, *BKCO1_3800026*, *UCRNP2_9053*, *UCDDS831_g00212*, *UCDDS831_g09295*, *UCDDS831_g08717*, *UCDDS831_g02163*, and *UCDDS831_g02126*). Genes encoding monooxygenase FAD (Flavin Adenine Dinucleotide)-binding protein (*MPH_09501*) and berberine family protein (*BKCO1_37000166*) were also identified (Cluster 2, Figure 7). Genes related to the hydrolysis of sulfates, such as arylsulfatases (*UCDDS831_g03540*) were also identified (Cluster 1, Figure 7). Moreover, genes encoding salicylate hydroxylase (*BKCO1_3300092*), tyrosinase, (homo)gentisate dioxygenase (HGD), and fumarylacetoacetate hydrolases (FMH) (*BKCO1_200014*, *UCDDS831_g07786*, *BKCO1_400034*, *UCRNP2_10214*, and *UCRNP2_9412*), which are responsible for the degradation of salicylic acid and tyrosine/phenylalanine, were also found to be differentially expressed. In addition, genes encoding Nudix proteins were observed (*BKCO1_4500094* and *UCDDS831_g04159*).

## 3. Discussion

One-year-old plants of *V. vinifera* were inoculated with *L. theobromae* Bt105, and the progression of the lesion throughout the sampling points suggested that a successful infection occurred (Figure 1). For a better understanding of this host–pathogen interaction, a dual RNA-Seq approach, as described previously for other plant–pathogen systems [21,24], was performed to simultaneously detect pathogen and host responses.

As expected, pectinases such as pectate lyases, which are involved in pectin cleavage [32] and GHs, as well as glycosidic bonds breakdown in oligo- or polysaccharides including cellulose and hemicellulose [33], were among the pathways activated in *L. theobromae*. As reported previously, *Botryosphaeria* spp. and particularly *L. theobromae* [14,34,35] can degrade lignocellulose (laccases, cellulases, pectinases, and xylanases), which enable them to penetrate the host plant. Pectin is the main component of the middle lamella of plant tissues and therefore has a fundamental role blocking intercellular fungal development. Pectin degradation seems to be important for fungal pathogenicity. It has been described that pectin-degrading enzymes are the most abundant plant cell wall modifying proteins expressed during infection in grapevines by fungi that cause grapevine trunk diseases (e.g., *Diplodia seriata*, *Neofusicoccum parvum*, and *Eutypa lata*) [36].

After the successful penetration of the fungus inside the host, the pathogen needs to control the stress originated by the new environment and overcome host’s defenses. Several genes related to secondary metabolism such as the cytochrome P450 monooxygenases superfamily, flavin-containing monooxygenases, and berberine enzymes-coding genes were identified. P450 monooxygenases are essential for fungal defense against antimicrobial substances produced by the hosts, which are involved in the production of mycotoxins, as aflatoxins and gibberellins, facilitating fungal adaptation to different niches [37,38]. Flavin-containing monooxygenases are microsomal proteins involved in the process of xenobiotics’ metabolism [39]. Berberine enzymes form a subgroup of the superfamily of FAD-linked oxidases, and in fungi, they are known to be associated with oligosaccharide oxidation that originates from the breakdown of the cell wall components [40]. We observed a decrease in the gene expression of *L. theobromae* berberine enzymes throughout the infection time (Cluster 2, Figure 7), suggesting that *L. theobromae* focuses its initial efforts in the degradation of toxic compounds produced by the plant and the degradation of the plant cell wall. On the other hand, the increase in gene expression of arylsulfatases may suggest that *L. theobromae* uses sulfur as an alternative strategy to fungal development during host penetration. Arylsulfatase plays an important role in the cycle of sulfur, allowing the development of microorganisms in hostile environments [41]. Moreover, the expression of genes encoding salicylate hydroxylase, tyrosinase, HGD, and FMH support the observations of Paolinelli et al. [42], who showed that in the presence of the host (grapevine), *L. theobromae* strain UCD256Ma has the ability to degrade salicylic acid and phenylpropanoid pathway precursors, which are produced by the plant host as defense mechanisms.

The biological role of genes of Nudix proteins is poorly understood, but some studies demonstrate that these effectors are capable of manipulating the host defense, leading to an increase of the plant susceptibility and contributing to the virulence of the fungus [43]. Several proteins related with Nudix proteins were also identified in the transcriptome and proteome of *L. theobromae* LA-SOL3 at 25 °C and 37 °C as having relevant functions for pathogenicity [44].

The most abundant functions of *V. vinifera* DEGs inoculated with *L. theobromae* were indicative of a host actively trying to combat infection (Figure 3; Figure 4). We observed the differential expression of many genes related to defense responses related to infection processes. When a grapevine is under fungal attack, it activates inducible defense mechanisms to effectively combat the invasion [45,46]. One of the inducible plant defense responses is the synthesis and secretion of PR proteins, which are known as being part of systemic acquired resistance. Most PR proteins have antimicrobial activities and toxicity toward pathogen cell walls. PR proteins can also be involved in plant defense signaling [47]. We identified many up-regulated PR-transcripts in infected plants. PR-3 and PR-4 are involved in the degradation of fungal cell walls, and PR-5 are involved in antifungal and membrane-permeabilizing activity [48]. Jayasankar et al. [49] showed that the inhibition of spore germination and hyphal growth of the fungal pathogen *Elsinoe ampelina* involves grapevine’s PR-5 (VvTLP-1). The antifungal activity of grapevine’s TLPs on the growth of *Diaporthe ampelina* and *Botrytis cinerea* mycelia was confirmed by Monteiro et al. [50]. The up-regulation of a hypothetical endo-1,3(4)-β-glucanase 2 coding gene (Cluster 6, Figure 5) might be related with PR-5. In fact, it has been reported that the functions of PR-5 and PR-2 against pathogenic microorganisms are related in some cases to endo-β-1,3-glucanase activity [51]. Derckel et al. [52] observed the up-regulation of PR-2 and accumulation of β-1,3-glucanase in grapevines during infection with *B. cinerea*. Moreover, we identified many genes encoding PR-10-related proteins in the *V. vinifera* transcriptome, which may also suggest a role in the defense against *L. theobromae*. As reported by Dadakova et al. [53] on *V. vinifera* cell’s suspension inoculated with *B. cinerea*, PR-10 proteins cause the inhibition of hyphal growth and the reduction of spore germination through membrane permeabilization mechanisms and interact with pathogen receptors. Lastly, Giacomelli et al. [54] also reported the expression of PR-12 transcripts in grape tissues infected with *B. cinerea*, suggesting a role in the defense. As reported for other Botryosphaeriaceae species, such as *N. parvum*, *L. viticola*, *Diplodia mutila*, and *D. seriata* [55], the expression of genes coding for PR proteins in grapevine is significantly intense within a short term after the first contact with pathogens.

Phytoalexin biosynthesis genes, such as PAL, STS, and CHS, were among the transcripts activated in the stem upon *L. theobromae* infection, suggesting that they could play a role in defense signaling. Infection of *V. vinifera* by *B. cinerea* activates the JA/ET pathway and induces genes related to phytoalexin biosynthesis. Phytoalexins are involved in the reduction of spore germination and inhibition of the fungus penetration [45,56,57]. Phenylalanine ammonia-lyases are key enzymes of phenylpropanoids metabolism. These enzymes are important in plant development and serve as markers for induced resistance in plants [58]. Stilbene synthases and CHS are members of the plant polyketide synthase superfamily, which is also involved in the signaling of defence responses and antifungal compounds production [59,60]. In grapevines with Botryosphaeria dieback, the induction and accumulation of stilbene compounds after 2 dpi was reported in *V. vinifera* cv. “Cabernet Sauvignon”, “Merlot”, “Ugni-Blanc”, and “RV4 hybrid” one-year-old plants^54^. Similar observations in one-year-old *V. vinifera* cv. “Cabernet Sauvignon” clone 19 in response to *N. parvum* infection were reported by Massonnet et al. [24] at 1 dpi. The expression data showed that the genes involved in JA biosynthesis were rapidly induced upon infection with *L. theobromae* (e.g., Clusters 3 and 6, Figure 5). Similar results were observed in peach trees (*Prunus persica* L.) inoculated with *L. theobromae* [27] and in *Arabidopsis thaliana* infected with *B. cinereal* [61,62].

Overall, our data demonstrated that distinct components of the phenylpropanoid pathway were activated, suggesting that when *L. theobromae* infects unstressed grapevines, it also selectively degrades pectin, which allows it to grow in intercellular spaces. *Lasiodiplodia theobromae*’s presence seems to trigger the host defensive mechanisms mediated by the activation of the phenylpropanoid pathway. However, host defense can be impaired by the activity of fungal phenylpropanoid precursors. On the other hand, some authors suggest that in the presence of the pathogen, the host defensive mechanism could be mediated through salicylic acid, and the plant defenses could be impaired by the activity of salicylic acid precursors [42]. Thus, variations of grapevine genotypes in relation to their resistance to infection, degree of fungal colonization, and severity of disease could be linked with this defense strategy.

In addition, genes related to Ca^2+^ signaling pathways were also activated, which is indicative of defense signaling. Ca^2+^-dependent protein kinases are triggered by pathogen-associated molecular patterns (PAMPs) via PRRs through changes in Ca^2+^ concentration within the cell. After Ca^2+^ signaling, Ca^2+^-dependent protein kinases activate Rboh through Ca^2+^ increase in the cytoplasm, Ca^2+^ binding, and phosphorylation [63]. The activation of Rboh induces reactive oxygen species (ROS), which trigger a hypersensitive response (HR) and cell wall reinforcement [64,65]. In addition, the activation of CDPK by intracellular effector proteins will trigger the biosynthesis of SA, JA, and ET through regulatory gene induction or enzyme activation such as the phenylalanine ammonia lyase mentioned above. Recent studies showed the involvement of CaM/CML-related genes in the induction of HR, suggesting their importance in plant immunity [66]. In plant cells, CaM/CML interacts with transcription factors such as WRKYs that we also observed. Massonnet et al. [24] reported the expression of genes related to the Ca^2+^ signaling and WRKY family transcription factor that were shown to be involved in grape resistance against necrotrophic fungal pathogens and in the regulation of lignin deposition [67,68].

In a recent study, the transcriptional responses of green shoots from grapevine cultivar ‘Summer Black’ infected with *L. theobromae* over 4, 8, and 12 h post-inoculation (hpi) were analyzed [23]. It is known that there is a broad range of variability in the aggressiveness of different isolates of *L. theobromae* against grapevines [1]. In fact, the *L. theobromae* strain used by Zhang et al. [23] successfully infected grapevine green shoots and produced visible lesions within 24 hpi. However, in our study at 1 dpi, there were no visible lesions produced by the *L. theobromae* strain used. In agreement with the lack of visual symptoms, at 1 dpi, we did not observe DEGs, which may be related to the different aggressiveness of both strains. Nonetheless, using a different cultivar and a different fungal strain, along with a longer time course (up to 10 dpi), our observations corroborate those of Zhang et al. [23], namely in what concerns hormone signal transduction and phenylpropanoid biosynthesis pathways. Both studies contribute with an in-depth view of the molecular interactions between grapevine and *L. theobromae* over time. In addition, we identify potential mechanisms used by *L. theobromae* during infection and establishment in the host.

## 4. Materials and Methods

### 4.1. Biological Material and Experimental Design

The experiment was conducted on 1-year-old grafted grapevine cuttings cultivar “Touriga Nacional” (rootstock 1103 Paulsen), which were propagated in 10 × 10 cm pots 2 months before inoculation, as described by Travadon et al. [69]. *Lasiodiplodia theobromae* strain Bt105 (isolated from *V. vinifera* cv. Castelão in Portugal) was grown on potato dextrose agar (PDA) (Merck, Germany) at 25 °C for 5 days prior to inoculation. In total, 24 plants were arranged in a randomized design in a greenhouse. Grapevines were subjected to two different treatments: inoculated and “mock” inoculated plants (control). The time of experiment was 10 days and samples were collected at 1, 3, 7, and 10 days post-inoculation (dpi). Sampling points were selected based on preliminary observations of plant symptoms, namely necrosis development and progression. Three biological replicates per treatment and per sampling point were used. Plants were disinfected with 96% ethanol between the second and third internode and cut with a cork borer; then, a colonized agar disc (4 mm) was placed on the wound, which was then covered with water-soaked cotton and sealed with Parafilm. Control plants were inoculated with discs of non-colonized PDA medium.

### 4.2. Sample collection

Green shoots (n = 3) were collected for each treatment for each sampling point. Three cm^2^ of stem tissue was harvested per inoculation site (1.5 cm of stem tissue below and above the inoculation site). Harvested material was immediately frozen in liquid nitrogen and subsequently stored at −80 °C for RNA extraction. Photographs were taken during the experiment, and the progression of the necrosis was monitored.

### 4.3. RNA Extraction, Library Preparation, and Sequencing

Stem tissue samples (n = 24) were ground in liquid nitrogen, and the total RNA was extracted using cetyltrimethyl ammonium bromide (CTAB)-based extraction protocol according to Gambino et al. [70] followed by a DNase I treatment (RNase-Free DNase Set, Qiagen). The concentration and quality of the total extracted RNA were checked using the ‘Quant-it ribogreen RNA assay’ (Life Technologies, Grand Island, NY, USA) and the RNA 6000 nano chip (Agilent Technologies, Santa Clara, CA, USA), respectively. Afterward, samples were stored at −80 °C until sequencing library preparation. Illumina mRNA sequencing libraries were made from 200 ng of total RNA of each sample using the QuantSeq 3′ mRNA-Seq Library Prep Kit (Lexogen, Vienna, Austria) according to manufacturer’s protocol using 15 cycles for the enrichment PCR step. Libraries were quantified by qPCR on a Lightcycler 480 (Roche, Basel, Switzerland), according to Illumina’s protocol ‘Sequencing Library qPCR Quantification protocol guide’, version February 2011. A high-sensitivity DNA chip (Agilent Technologies, Santa Clara, CA, USA) was used to control the library’s size distribution and quality. The libraries were equimolarly pooled and sequenced at the NXTGNT facility (www.nxtgnt.ugent.be, Ghent University) on an Illumina NextSeq 500 sequencer controlled by NextSeq Control Software (NCS 2.1.2) using a High Output Kit v2 (75 Cycles), generating single-end 75 bp reads.

Per sample (n = 24), on average, 20.5 ± 4.7 million reads were generated. Adequate sequencing quality was confirmed using FastQC version 0.11.5 [71]. Afterwards, these reads were trimmed using Cutadapt [72] version 1.11 to remove the “QuantSEQ FWD” adaptor sequence. Potential contamination was checked using FastQ Screen version 0.7.0 [73]. The trimmed reads were mapped to the combined annotated reference genome of *V. vinifera* (NCBI Reference Sequence: GCF_000003745.3) and *L. theobromae* strain LA-SOL3 (SAMN08892999, [44]) using the STAR aligning software version 2.5.3a [74]. Following the mapping step, the RSEM software version v1.2.31 [75] was used to generate the count tables for both *V. vinifera* and *L. theobromae*. Two samples of control plants collected at time point 3 dpi were removed after the detection of *L. theobromae* contamination. Sequencing and mapping quality reports were aggregated with MultiQC version 1.0 [76]. Raw reads and raw count data are accessible in the Gene Expression Omnibus (GSE129109).

### 4.4. Pairwise Comparison of the Samples Based on V. vinifera Genes

For each time point, differential gene expression analysis was done between the samples of the “mock” inoculated plants as the control group and the samples from the inoculated plants as the treatment group using edgeR [77] performing the following steps. First, read counts were normalized using the standard edgeR [77] normalization method. Only genes with a count per million (cpm) >1 in at least three samples were considered for statistical differential gene expression analysis. Subsequently, a general linear model was built, and statistical testing was done using the empirical Bayes quasi-likelihood F-test. Computed *p*-values were adjusted using the Benjamin–Hochberg false discovery rate (FDR) correction to account for multiple comparisons. Genes having an FDR < 0.01 and a log_2_ fold change (FC) > 1 or <−1 were considered significantly differentially expressed. To understand the functions of the differentially expressed genes (DEGs), a Gene Ontology (GO) analysis was performed using the GOslim software. The significant enrichment of GO terms based on hypergeometric distribution followed by FDR <0.05 correction was used for comparison between the test set and reference set of each species using ShinyGO v0.61 (http://bioinformatics.sdstate.edu/go/).

### 4.5. Time Series Analysis

In addition to the pairwise comparisons, a time series analysis with the maSigPro R package [78] was performed. Read counts of *V. vinifera* were normalized using the standard edgeR [77] normalization method. Only genes with a count per million (cpm) > 1 in at least three samples were considered for the time series analysis. A full general linear model with all these genes, all conditions (treatment and control), and with a linear and quadratic time factor was fitted to the data. Differential expression analysis was performed whereby genes were significantly differentially expressed if one or more conditions/factors have a significant contribution to the fitted model using an FDR < 0.05. For each of these temporal DEGs, a stepwise regression was performed modeling the relationship between expression and the time points, whereby the inclusion or exclusion of the quadratic time factor was decided based on a *p*-value of 0.05. Genes with a regression model with an R^2^ > 0.8 were further used in a Ward’s hierarchical clustering analysis. In the clustering analysis, genes with a similar expression profile (i.e., a similar regression model) were clustered together.

In addition, for the *L. theobromae* genes, a time series analysis was performed as described above. Thereby, a full general linear model with only a linear and quadratic time factor was fitted to the data.

## 5. Conclusions

**Figure 8:**
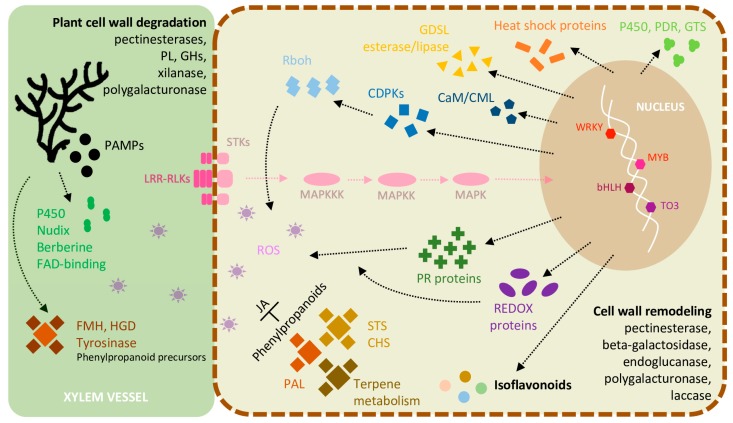
Model illustrating the most frequent molecular events occurring in *V. vinifera* infected with *L. theobromae*. Transcriptomic analyses highlight the ability of *L. theobromae* to degrade cell wall components and to control host defences. The perception of the fungus by grapevine trigger many inducible defence mechanisms, signaling cascades, and stress response. PL: pectate lyase; GHs: glycoside hydrolases; PAMPs: pathogen=associated molecular patterns; LRR-RLKs: leucine-rich repeat receptor-like kinases; STKs: receptor-like serine/threonine-protein kinases; MAPKs: mitogen-activated protein kinases; FMH: fumarylacetoacetate hydrolase; HGD: (homo)gentisate dioxygenase; Rboh: NADPH oxidases respiratory burst oxidase homologs; CDPKs: Ca^2+^-dependent protein kinases; CaM/CML: Calmodulin/Calmodulin-like proteins; PDR: pleiotropic drug resistance; GTS: glutathione transferase; PR proteins: pathogenesis-related proteins; JA: jasmonic acid; STS: stilbene synthase; CHS: chalcone synthase; PAL: phenylalanine ammonia lyase; Transcription factors: WRKY, MYB, bHLH, TO3.

The outcomes of this study provide the first report that assess both host and pathogen transcriptomes simultaneously, detecting *L. theobromae*-specific transcripts in the same sample, such as genes related to phenolic compounds, carbohydrate metabolism, transmembrane transport, and toxin synthesis. In addition, genes encoding for the degradation of plant phenylpropanoid precursors such as salicylate hydroxylase, tyrosinase, (homo)gentisate dioxygenase, and fumarylacetoacetate hydrolases were detected, suggesting that *L. theobromae* could evade the host defense response using the phenylpropanoid pathway. Additionally, our results corroborate other studies focused on grapevine transcriptome when inoculated with *L. theobromae* as genes related to phytoalexin biosynthesis, hormone metabolism, cell wall modification enzymes, many pathogenesis-related proteins, and phenylpropanoid compounds involved in the host responses to infection. An integrated overview of the most frequent responses detected is summarized in Figure 8 (inspired on the model of *V. vinifera* and the bacterium *Xylella fastidiosa* interactions [79]). Comparative studies with other cultivar or pathogen strains differing in susceptibility and aggressiveness might give a crucial complement to understand the plant resistance/susceptibility to infections. Moreover, further studies focused on systemic acquired resistance and induced systemic resistance (“priming”) are necessary to understand the mechanisms of fast, strong, and effective defense responses after pathogen challenge.

## Supplementary Materials

Supplementary Materials can be found at https://www.mdpi.com/1422-0067/20/23/6083/s1. Figure S1: Saturation curves of *V. vinifera* genes, Figure S2: Saturation curves of *L. theobromae* genes, Table S1: Gene Ontologies of DEG’s in response to *L. theobromae* inoculation in *V. vinifera* at 3 dpi, Table S2: Gene Ontologies of DEG’s in response to *L. theobromae* inoculation in *V. vinifera* at 7 dpi, Table S3: Gene Ontologies of DEG’s in response to *L. theobromae* inoculation in *V. vinifera* at 10 dpi, Table S4: Significantly enriched GO terms identified from differentially expressed *V. vinifera* genes, Table S5: Gene clusters of DEG’s of *V. vinifera* with a regression model with an R_2_ > 0.8 during the colonization of grape stems, Table S6: Gene Ontologies of DEG’s of *L. theobromae* over time, Table S7: Gene clusters of DEG’s of *L. theobromae* with a regression model with an R_2_ > 0.8 during the infection of grape stems.

## Abbreviations

dpi	Days post-inoculation
hpi	Hours post-inoculation
GO	Gene Ontology
DEGs	Differentially expressed genes
PR	Pathogenesis related
PRRs	Pattern recognition receptors
GSTs	Glutathione transferases
PDR	Pleiotropic drug resistance
TLPs	Thaumatin-like proteins
STS	Stilbene synthase
CHS	Chalcone synthase
PAL	Phenylalanine ammonia lyase
ET/JA	Ethylene/Jasmonic acid
CDPKs	Ca2+-dependent protein kinases
FAD	Flavin adenine dinucleotide
CaM/CML	Calmodulin/Calmodulin-like transcripts
CTAB	Cetyltrimethyl ammonium bromide
