# Supplementary figures and images for: Dual RNA Sequencing of Vitis vinifera during Lasiodiplodia theobromae Infection Unveils Host–Pathogen Interactions
Source: Int J Mol Sci. 2019 Dec 3;20(23):6083. doi: 10.3390/ijms20236083 (PMC6928909; doi:10.3390/ijms20236083)

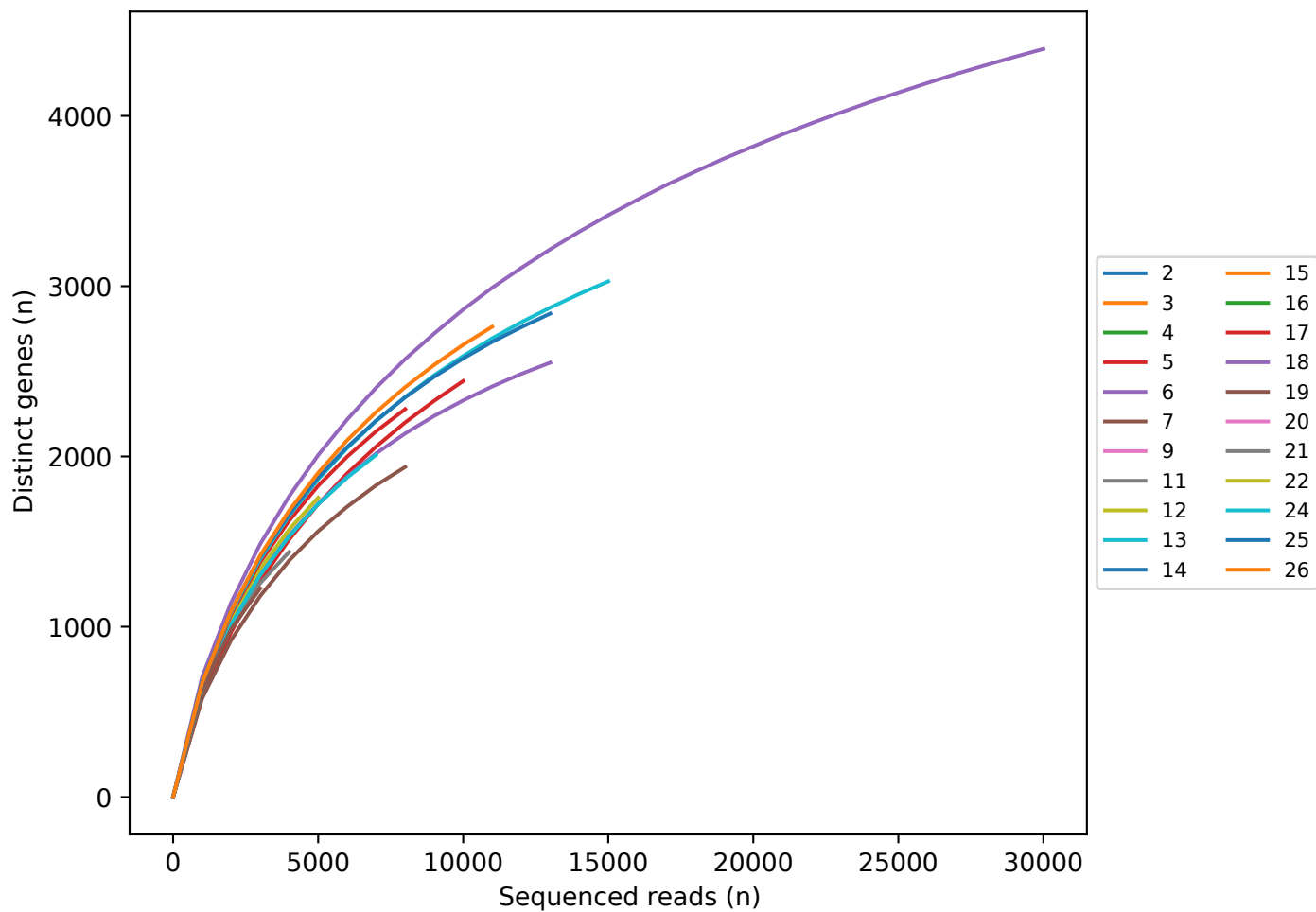

Supplement: Supplementary file 1 [file ijms-20-06083-s001.zip › Tables/Supplementary_Figure_S2_28-03-19Saturation2_V3.pdf]

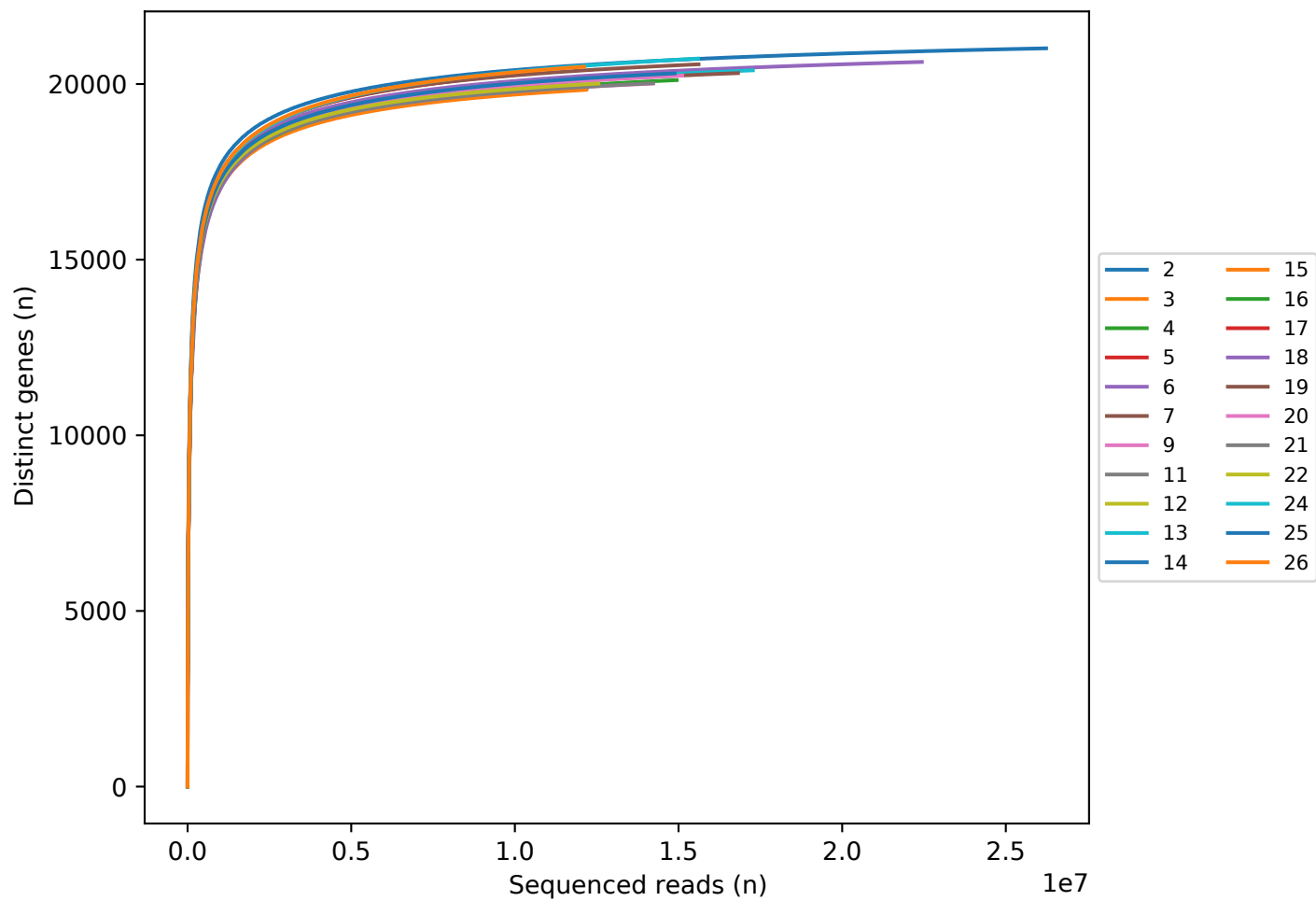

Supplement: Supplementary file 1 [file ijms-20-06083-s001.zip › Tables/Supplementary_Figure_S1_28-03-19Saturation2_Grape.pdf]
